# Effect of PLA Active Packaging Containing Monoterpene-Cyclodextrin Complexes on Berries Preservation

**DOI:** 10.3390/polym13091399

**Published:** 2021-04-26

**Authors:** Friné Velázquez-Contreras, Nelsy García-Caldera, José Daniel Padilla de la Rosa, Domingo Martínez-Romero, Estrella Núñez-Delicado, José Antonio Gabaldón

**Affiliations:** 1 Department of Health Sciences, Campus de los Jerónimos, Universidad Católica San Antonio de Murcia, No.135 Guadalupe, 30107 Murcia, Spain; fvelazqu@up.edu.mx (F.V.-C.); enunez@ucam.edu (E.N.-D.); 2 Escuela de Administración de Instituciones (ESDAI), Universidad Panamericana, Álvaro del Portillo 49, Ciudad Granja, Zapopan 45010, Mexico; ngarciac@up.edu.mx; 3 Departamento de Tecnología Alimentaria, Centro de Investigación y Asistencia Tecnológica y Diseño del Estado de Jalisco (CIATEJ), Marcelino García Barragán 800, Guadalajara 44270, Mexico; jdpadilla@ciatej.mx; 4 Department of Food Technology, University Miguel Hernández, Orihuela, 03312 Alicante, Spain; dmromero@umh.es

**Keywords:** active packaging, poly (lactic acid), thymol, carvacrol, β-cyclodextrin, berries, shelf life

## Abstract

Blackberries and raspberries are highly perishable and fragile products, which limits their shelf life. The effect of biodegradable active packaging of blackberries and raspberries containing 2.5% and 5.0% weight (wt%) of thymol or carvacrol complexed in β-cyclodextrins (β-CDs), successively added to poly (lactic acid) (PLA), and melt-processed by injection molding was evaluated under stored conditions at 4 °C for 21 days, using as reference commercial clamshell and PLA package control samples. Thus, physicochemical, headspace, microbiological, and sensory quality studies were carried out in order to compare the efficacy of the different packages. Concerning weight loss, color, and total phenolic and soluble solids content, significant differences were detected when compared with commercial clamshell packaging. The results show that the PLA packages containing thymol and carvacrol complexes maintained the color, weight, and phenolic content of berries until day 21, with a score up to 45% better compared to commercial clamshell. The headspace analysis detected 101 mg L^−1^ (ppm) of thymol and 35 ppm of carvacrol on the first day of refrigeration; these concentrations decreased with time. This release mechanism of carvacrol and thymol into the PLA package modified the initial atmosphere composition. After 21 days of storage, the berries had 4.25 degrees of acceptance, without adverse perception of aroma or flavor for both carvacrol and thymol compounds. A general microbial inhibition was observed for yeast and molds, which increased with the concentration of monoterpene in PLA packages, and showed an inhibition of 3.5 log units for PLA packages containing thymol, and of 3 log units for those containing carvacrol. Overall results show that PLA/β-CD-thymol 5.0% packages prolonged raspberries’ and blackberries’ shelf life by one more week at 4 °C, compared with commercial clamshell packaging.

## 1. Introduction

Since ancient times and before the start of agriculture, berries have been valuable as a primary food source for humans and other primates, and their consumption has increased greatly in recent years. This marked trend could be related to health as a recent motivational purchase vector of consumers since—despite berries having a number of highly appreciated nutritional (they represent an important source of macro- and micronutrients) and organoleptic attributes such as their sharp color, exclusive flavor, and delicate texture—their health-promoting properties have been largely associated with their high levels of bioactive compounds (including anthocyanins, ascorbic and phenolic acids, and flavonoids) with known benefits in the prevention of certain types of cancer, as well as heart and age-related diseases, such as neurodegeneration [1].

However, berries have a short storage life (about 3 weeks at low temperature) as a result of their high respiration and water loss, which results in the fruit shriveling and softening. In addition, they show high susceptibility to mechanical damages and decay, which is generally increased by postharvest fungal infections (mainly caused by *Botrytis cinerea* and *Colletotrichum gloeosporioides*). Hence, berries should be marketed soon after picking, since they are very perishable fruits [2], and must be rapidly cooled and kept at temperatures close to 0 °C and 90–95% of relative humidity (RH) [3] for maximum postharvest preservation, since temperature management is the most critical postharvest factor [4].

Blackberry and raspberry (*Rubus* spp.) are considered non-climacteric fruits; they must be harvested at or near full maturity, since habitually they will not continue to ripen once isolated from the plant. Therefore, after harvest, attention to detail is required throughout the handling system between the sites of production and consumption, since fruits are living organs subject to continuous changes. Some of these changes are desirable and may require specific actuation protocols (including treatments and/or techniques) to promote them, while others, such as ripening and senescence, the last stages in the development of fresh berry fruits, which are characterized by some irreversible processes that lead to breakdown and death of the fruit, are undesirable and require the application of corrective actions to delay and minimize their occurrence and severity [5]. Therefore, understanding the biological and environmental factors affecting the postharvest deterioration rate (respiration, ethylene evolution, compositional changes, temperature, CO_2_, and pathogens incidence) is crucial to the implantation of suitable strategic actions for maintaining quality and extending berries’ postharvest shelf life.

In this context, different approaches have been investigated to extend the shelf life of these fruits, with the application of modified atmosphere packaging (MAP) being widely used. In this preservation technique, when the air surrounding the berries in the package is changed to another composition, close to 10% O_2_ and 15% CO_2_, the decay is significantly retarded, and the fruits show a more attractive color compared with those stored under standard conditions [6]. In addition, MAP promotes microbial load reduction, since berries keep respiring the trapped air until the concentration of CO_2_ swiftly reaches the critical level (10–15%) required to inhibit microorganism growth, such as *Botrytis cinerea*. However, some negative effects include the slow-down of the cooling of the packaged products and an increased potential for water condensation within the package, as well as lower respiration and softening rates that have been described in this type of packaging, which consequently reduces the berries’ shelf life. Therefore, appropriate postharvest techniques are needed, as well as a low storage temperature to maintain the quality of the fruits, to prevent physical injury, and to keep diseased or wounded berries out of packages, as rot can rapidly spread from diseased berries to the nearby healthy ones. In addition, temperature fluctuations that can occur during storage, transport, and retail display can generate an unfavorable atmosphere inside the package [7] and affect the desirable flavor and aroma organoleptic parameters [8,9].

Due to the increasing consumer awareness about residues of agrochemicals, a set of limitations on their use was imposed by many countries. However, the consumption and often uncontrolled disposal of single-use plastic packaging is regarded as one of the main environmental hazards [10]. Indeed, the forecast by 2050 is not optimistic, since oceans could contain more plastic than fish by weight, and the impacts of degraded or intentionally produced microscopic-sized plastics on human health remain unknown [11]. In fact, plastic pollution is considered to be among the greatest challenges the world faces.

Thus, to respond to growing consumer demand, some of the strategic approaches recently outlined by the European Commission under the Food 2030 umbrella are focused on the development of impactful solutions to the urgent, complex, and interconnected challenges inherent to food systems, applying the bioeconomy principles. Challenges in this area include, among others, achieving zero food waste, promoting the employment of safer alternatives to replace synthetic pesticides, and rethinking food packaging for better biodegradable options that limit micro plastics and greenhouse gases release [12,13].

Currently, most bioplastics are produced from agricultural crop-based feedstocks, [14] like cellulose, proteins, starch, renewable polyethylene and polyvinyl chloride (PVC), polyhydroxyalkanoates (PHAs), or poly (lactic acid) (PLA). Among them, PLA exhibit good functional properties in terms of bio-absorbability, biodegradability, biocompatibility, mechanical strength, and transparency, and meet several packaging requirements to extend the shelf life and protection of these types of fruits [15]. Moreover, PLA could be enhanced with antimicrobial activity via the incorporation of natural essential oils, displaying a promising active packaging in view of reducing postharvest losses, and retaining high standards in terms of environmental sustainability [16].

The main disadvantage of using essential oils are their highly volatile nature, as they are quite sensitive to light, insoluble in water, and have an unpleasant aroma, which limits their application in active food packaging [17]. For this reason, the search for strategies that protect the active compounds from volatilization during processing, and prevent a fast release of these kinds of compounds, is required. Thus, the introduction of nanoparticles that include essential oils or their main components as a filler into biopolymer-based materials could be a successful approach to fulfil these drawbacks.

Based on this concept, recent studies have reported the antimicrobial effectiveness by in vitro tests of PLA and starch films with cinnamaldehyde against *Escherichia coli* and *Listeria Innocua* through [18]; or against *Escherichia coli* and *Staphylococcus aureus* by PLA nanofilms incorporating inclusion complex of cinnamon essential oil in *β*-cyclodextrins [19]. In addition, fungal growth hurdles of *Alternaria alternata* were shown to be effective, using as additives up to 10 wt% of thymol or carvacrol complexed in β-cyclodextrins, which were successively added to PLA and melt-processed by injection molding [20].

Thus, the development of PLA active packaging containing the above described materials is an innovative antimicrobial approach in the agri-food sector [16], and in particular to protect blackberries and raspberries during their storage and distribution to improve their shelf life by reducing bacterial and fungal growth [21]. In this type of packaging, one of the most common approaches is based on the release of antimicrobial compounds from the package. Although these compounds could be of synthetic or natural in origin, due to the consumer’s growing refusal of synthetic additives in food, the use of natural antimicrobial substances could be promoted.

Carvacrol and thymol are two monoterpenoid compounds naturally present in some plant species such as oregano and thyme, which have shown a wide range of activity against pathogenic microorganisms, including Gram-negative and Gram-positive species [22]. Due to their hydrophobic nature, the presence of a free hydroxyl group, and a delocalized electron system, which are responsible for the disruption of the cytoplasmic membrane of bacterial cells, disturbing their functional properties, causing leakage of integrity and intracellular material [23], they are also effective against various fungi such as *Aspergillus* spp., *Botrytis cinerea*, *Fussarium* spp. [22], or *Penicillium* spp., [24], by inducing envelope damage and blocking ergosterol biosynthesis.

Recently, Zhang et al. [25] demonstrated that concentrations of thymol between 65 mg L^−1^ and 100 mg L^−1^, and of carvacrol between 120 mg L^−1^ and 140 mg L^−1^, showed high postharvest efficacy against *Botrytis cinerea*, changing the morphology of the cell wall, breaking the membrane by collapse, thus causing deformation and deterioration of hyphae and conidia. In addition, thymol and carvacrol were complexed in native and modified β-cyclodextrins in a previous study, and successfully applied by in vitro tests against filamentous fungi [26] and bacteria [17].

The two cyclodextrins and two monoterpenes to be added to PLA have been classified as generally recognized as safe (GRAS) by the U.S. Food and Drug Administration (FDA), and included in the list of additives permitted for direct addition to food for human consumption.

In this context, the antifungal effect of a controlled release system of thymol and carvacrol encapsulated in β-cyclodextrin, incorporated into a biodegradable polymeric matrix of PLA by industrial injection, was previously assessed in vitro [20]. At present, there are very few research results on their efficacy in extending the shelf life of fresh produce in vivo under storage conditions. The aim of this study was to evaluate the in vivo effect of the biodegradable packaging in order to enhance the quality attributes of berries during refrigerated storage, to increase their shelf life in commercial distribution, trying to demonstrated the functionality of this active packaging.

## 2. Materials and Methods

### 2.1. Materials

Thymol (CAS: 89-83-8, 98.7% purity), carvacrol (CAS: 499-75-2, 99.5% purity), and β-cyclodextrin (β-CDs > 95%, food grade) were provided by Sigma-Aldrich Corp. (Saint Louis, MO, USA).

For injection molding applications, a poly (lactic acid) (PLA, Ingeo™ ref. code: 3251D) biopolymer with required physicochemical parameters ($\bar{M}$w = 5.5 × 10^4^ g/mol; PI = 1.62 and 99% L-lactide/1% D-lactide isomers content) and flow capability performances [13] was purchased from PromaPlast Co. (Guadalajara, Jalisco, Mexico). Potato dextrose agar (PDA), plate count agar (PCA), and violet red bile agar (VRBA) were provided by Bioxon, Mexico. All other chemical products used were of analytical grade.

### 2.2. Packaging Preparation and Characteristics

In preliminary experiments, spray drying was used to prepare microcapsules of carvacrol and thymol complexed in *β*-cyclodextrins in solid state, which were directly mixed with PLA (up to 5 wt%) and extruded to produce pellets [20]. The microcapsules proved to act as plasticizers, being particularly able to reduce intermolecular forces of PLA chains, thus improving the breaking properties and stretchability, which were subsequently required for adequate melt-processing of the pellets by injection-molding to obtain (12 cm long × 10 cm width × 3.0 cm height and 0.1 cm wall thickness) boxes for the in vivo test. Briefly, from pellets containing as ingredients PLA (97.5% and 95% weight percentages, wt%) and dehydrated complexes of β-CD-carvacrol or β-CD-thymol (at 2.5% and 5% wt%), a representative number of boxes were obtained by injection of the pellets, which were identified as PLA/β-CD-carvacrol, 2.5% wt%; PLA/β-CD-carvacrol, 5.0% wt%; PLA/β-CD-thymol, 2.5% wt%; PLA/β-CD-thymol, 5.0% wt%. In addition, boxes containing 100% PLA were also formed and used as control.

### 2.3. Design and Application of Storage Tests

**Figure 1:**
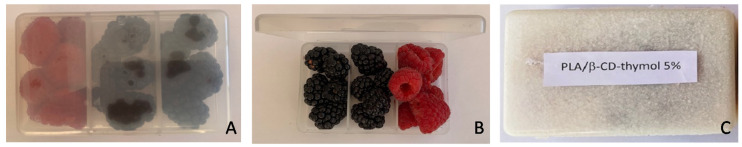
Boxed samples containing blackberries and raspberries. (**A**) PLA/C, closed; (**B**) PLA/β-CD-carvacrol, 2.5% wt%, open; (**C**) PLA/β-CD-thymol, 5.0% wt%, closed.

Blackberries (*Rubus ulmifolius* var. Dasha) and raspberries (*Rubus idaeus* var. Maravilla) were selected to assess the antimicrobial activity of the PLA/β-CD-carvacrol and PLA/β-CD-thymol packaging, as well as their effect on quality attributes of berries during refrigerated storage. Thus, commercial Driscoll’s packed samples from the western zone of Mexico, Jalisco state, were purchased in October 2020, in a local supermarket of Zapopan (Jalisco, Mexico), and transported in coolers to the Universidad Panamericana and CIATEJ (Center of Research and Assistance of Technology and Design of Jalisco State). After that, berries were stored at 4 °C ± 2 °C for six hours until use. Samples of 47 g (6 blackberries) and 25 g (6 raspberries) were weighed using a common scale in Adventurer Pro balance (OHAUS, Parsippany, NJ, USA) and placed inside the developed boxes (Figure 1), which were divided into six groups as follow:

- PLA/β-CD—thymol, 2.5% containing blackberries and raspberries
- PLA/β-CD—thymol, 5.0% containing blackberries and raspberries
- PLA/β-CD—carvacrol 2.5% containing blackberries and raspberries
- PLA/β-CD—carvacrol 5.0% containing blackberries and raspberries
- PLA/C (packaging control) containing blackberries and raspberries
- Commercial clamshell (control) containing blackberries and raspberries

For each group, thirty replicate packages were prepared to accomplish all physicochemical, microbiological, and chromatographic determinations at initial (T0), 7 days (T1), 14 days (T2), and 21 days (T3). The packages were stored at 4 °C ± 2 °C and 85–90% RH in a conventional refrigerator (Whirlpool Mod. WS5501Q).

### 2.4. Physicochemical Analyses

#### 2.4.1. Weight Loss

Weight loss of stored blackberries and raspberries was measured on day 0, and subsequently on the corresponding sampling days, in triplicate, using an Adventurer precision balance (OHAUS, Parsippany, NJ, USA), and expressed as percent of weight loss per initial fruit weight by means of the following equation:

$$\mathrm{WL}=\left(\frac{\left(m1-m2\right)}{m1}\right)\;\times \;100$$

where (m_1_) was the initial weight of the berries, and (m_2_) the weight after the corresponding storage time.

#### 2.4.2. Color

The surface color of blackberries and raspberries was determined by measuring at least six berries from each package, in triplicate, at the beginning and at the end of storage, using a colorimeter Konica Minolta (Chroma Meter, Tokyo, Japan). The colorimeter used the CIE standard illuminant D65 that represents daylight more completely and accurately than B and C illuminants [27]. Measurements were obtained using the CIE L*a*b*: L * indicates lightness, a * indicates chromaticity on a green (-) to red (+) axis, and b * indicates chromaticity on a blue (-) to yellow (+) axis. Results were expressed as the mean ± standard deviation of three determinations.

#### 2.4.3. Soluble Solids Content

The determination of soluble solids (SSC) was carried as described by Almenar et al. [28]. For this, approximately 20 g of blackberries and raspberries were blended in a mortar for 60 s. SSC in the obtained juice were measured using a digital handheld refractometer (Atago, Tokyo, Japan) at 20 °C. Three measurements were taken on each sample, and the results were expressed as °Brix.

#### 2.4.4. Total Phenolic Content

The total phenolic content (TPC) of the berries’ extract was determined colorimetrically using the Folin–Ciocalteu reagent, according to a modification of the López Miranda et al. [29] method. For that, the gallic acid curve was prepared by serial solutions from 1 mg mL^−1^ standard. The berries’ extracts were dissolved in ethanol 1 mg/mL (*v*/*v*). For each extract, an aliquot of 20 µL was added to 100 µL of Folin–Ciocalteu in spectrophotometer cuvettes and allowed to react for 5 min, then 80 µL of 7.5% sodium carbonate solution was added and homogenized. Then, it was incubated at room temperature in the dark for 2 h, and the optical density was measured at 750 nm using an Infinite 200 PRO Microplate Reader from TECAN. Data were expressed as gallic acid equivalents. Results were expressed as the mean ± standard deviation of three determinations.

### 2.5. Study on the Sustained Release of Carvacrol and Thymol

**Figure 2:**
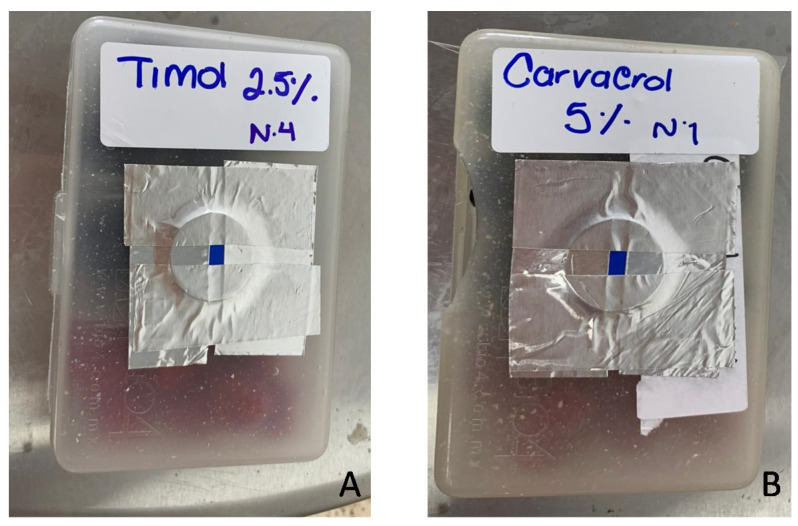
Boxes containing blackberries and raspberries adapted for headspace sampling. (**A**) PLA/β-CD-thymol, 2.5% wt%; (**B**) PLA/β-CD-carvacrol, 5.0%.

The release rate of carvacrol and thymol from the PLA were evaluated in the headspace by gas chromatography coupled to mass spectrometry (HS-GC-MS) using a GC/MSD System Agilent Technologies 7890B/5977A (Palo Alto, CA, USA). To perform these determinations, a representative number of berry containers were properly adapted to do correct sampling (Figure 2). Briefly, the PLA packages were drilled to make a hole through which to sample the terpene phenols released during storage. The hole was sealed with a PTFE/silicone septum (20 mm, Supelco) and fixed using reinforced aluminum adhesive tape. A gas-tight syringe A-2 Series (VICI Precision, Los Ángeles, CA, USA) was used to sample 1 mL of headspace through the septum in each package, and further introduced into the injector operated in split mode (3:1) at 240 °C. The separation was carried out on an INNOWAX capillary column (60 m × 250 µm × 0.25 µm, Folsom, CA, USA) from Agilent Technologies, and helium was used as carrier gas, at a flow rate of 0.8 mL min^−1^. The oven temperature was constant at 200 °C, held for 16 min. The MSD transfer line and ion source temperatures were operated at 260 °C and 250 °C, respectively, in electron-impact ionization mode (70 eV).

Carvacrol and thymol identification were performed by injection of pure standard (Sigma-Aldrich, Saint Louis, MO, USA) and spectral data from the NIST14 MS library database in a previous acquisition in full scan mode (*m/z* 29–300). Quantification was performed by using selected ion monitoring (SIM) focused on *m/z* 91, 135, and 150 characteristic ions of thymol and carvacrol. Chromatographic responses (peak area abundance) were monitored to obtain the comparative concentration of each compound in the studied samples. Three replicates for each group and sampling day were evaluated.

### 2.6. Microbial Analysis

Ten grams of the sample of each package (group) were ground and suspended in 90 mL peptone water (0.1% *v*/*v*), and the suspension was mixed in a blender (Seward Stomacher 400) for 5 min. Serial dilutions (10^−1^, 10^−2^, and 10^−3^) of the homogenated berries were plated on the surface of selective media: aerobic mesophilic bacteria (AMB) on plate count agar (PCA), incubated at 35 °C for 48 h; total coliforms (TC) using violet red agar (VRBA), incubated at 35 °C for 48 h; and yeast and molds (YM) on potato dextrose agar (PDA), incubated at 25 °C for 5 days.

All culture mediums were from BD Bioxon, México, and microbial analysis was performed in triplicate, according to the Official Standard Method guidelines [30]. The colony forming units (CFU) were expressed as log CFU (colony-forming units) per gram of blackberry and raspberry. Results were expressed at the mean ± standard deviation of three determinations.

### 2.7. Sensory Atributes Analysis

The organoleptic parameters—color, flavor, odor, texture, and overall impression of the blackberries and raspberries packaged in the control clamshell and control PLA, as well as in PLA packages containing β-CD-thymol (2.5% and 5.0% wt%) or β-CD-carvacrol (2.5% and 5.0% wt%)—were scored using the 5-point scale (where extremely like = 5, moderately like = 4, neither like nor dislike = 3, moderately dislike = 2, and extremely dislike = 1) by a panel of 30 judges (15 men and 15 women), with experience in sensory analysis (all members of the academy of sciences at Universidad Panamericana) by means a hedonic analysis [31]. The samples were delivered coded with 3-digit numbers, in sufficient quantity at an adequate temperature (21 °C), in order to minimize the variability associated with this type of non-descriptive hedonic test. In addition, all evaluations were carried out at 0, 7, 14, and 21 days of storage, in individual cabinets that fit the design standard specifications. As consensus of opinion among judges, the end of shelf life was set for a score of 3 or less for any of the organoleptic characteristics evaluated. Results were expressed as the mean ± standard deviation of three determinations.

### 2.8. Statistical Data Treatment

Experiments were carried out in a randomized design, in triplicate, and the data were reported as means ± 1 standard deviation. When ANOVA was significant (*p* < 0.05), means were separated by Turkey’s range test. The statistical analyses were performed using 01Statgraphics Centurion XV.

## 3. Results and Discussion

To investigate whether the developed active packaging releasing carvacrol or thymol would prevent deterioration of non-climacteric berries, storage stability was studied.

### 3.1. Weight Loss

**Figure 3:**
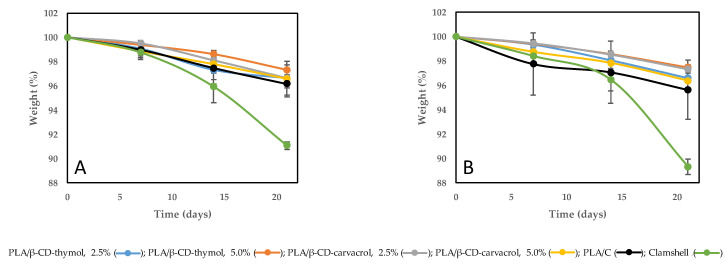
Effect of type of packaging and storage time (0, 7, 14, 21 days) at 4 °C on the blackberries’ (**A**) and raspberries’ (**B**) weight losses (expressed as percentage).

Usually, postharvest moisture loss alters fruit appearance, flavor, and texture, and reduces its marketable weight [32]. Since raspberries and blackberries are prone to dehydration due to the lack of epicuticular wax, a maximum moisture loss of 6% is considered as commercially acceptable [32]. Figure 3 shows the weight loss of control and active packaged blackberries (A) and raspberries (B) samples with storage time.

As can be seen, the weight loss in raspberries (Figure 3B) was significantly greater (*p* < 0.05) than for blackberries (Figure 3A). This fact could be due to the mass transport and diffusion of water vapor that happens during the physiological processes; raspberries have higher respiration rates (metabolic activity 49 mg CO_2_ kg^−1^ h^−1^ at 10 °C) than blackberries (metabolic activity 31 mg CO_2_ kg^−1^ h^−1^ at 10 °C) [33].

In addition, in the clamshell control samples (see Figure 3), a higher moisture loss of berries stored at 4 °C was observed at the end of the study (*p* < 0.05): 9% for blackberries and 11% for raspberries, both of which are higher than the commercially acceptable value of 6%.

This behavior was attributed to excessive water loss, from dry matter consuming water, and we should not be amazed, since in the fresh market, berries are commonly packed in clamshells with opening ratios between 3% and 10%, preventing ethylene, heat, and moisture accumulation [8]. However, the vents could make berries with a wide surface area ratio (like proposed here) more susceptible to freezing, chilling, and drying damage [34]. Similar moisture losses, or still higher values that are directly proportional with the diameter and number of opening ratios, have been described in the literature [35]. To overcome commercial clamshells’ limitations, the active packaging here proposed (with lid closed), which has low water vapor permeability between fruits and the surrounding air, retains a relative humidity inside the package [36]. Therefore, the observed weight losses are even 7% lower in the berries inside the control packaging (PLA/C).

Taking into account the above described commercially acceptable moisture loss value for berries, all PLA active packaging containing monoterpene-cyclodextrin complexes showed a slower weight loss trend, reaching, in the worst case, a decrease of 3% at the end of the study. The higher reduction of weight loss by carvacrol or thymol would indicate a role of these compounds in lowering the dehydration process [35]. PLA β-CD-thymol 5.0 wt% showed the lowest weight loss for blackberries and raspberries, with a significant difference (*p* < 0.05) when compared to PLA/C and commercial clamshells control samples, stored under refrigeration conditions at 4 °C after 21 days of assay. Moreover, berries in commercial clamshells were unsaleable by day 16, with a weight loss of 6%.

### 3.2. Color

The development of the preferred red/purple-bluish color in berries is mainly due to anthocyanins, and during postharvest ripening of the fruit, numerous changes occur in pigments affecting color [37]. As can be seen in Table 1, color changes were significantly affected in blackberries as well as in raspberries (see Table 2) stored under refrigeration at 4 °C, varying by type of packaging and storage time.

The ANOVA test showed significant differences (*p* < 0.05) with respect to storage time. In the case of blackberries, the packaging treatments’ decreasing lightness (L*) and modified chromatic coordinates a* and b*, increasing the red shift (a*) as well as a yellow color trend (b*), were observed in the clamshell with respect to the PLA β-CD-thymol 2.5 wt% and PLA β-CD-thymol 5.0 wt% packaging, respectively.

With respect to the raspberries, color changes were significantly affected by packaging treatments, decreasing lightness (L*), and chromatic coordinates (a* and b*). However, the raspberries packed in PLA β-CD-thymol (2.5 or 5.0 wt%) showed less loss of red color (a*). This could be due to the protective and antioxidant effect of a sustained and controlled release of encapsulated thymol inside the PLA package, as has been previously described for table grapes stored for 56 days under modified atmosphere packaging with thymol [38].

In summary, a decrease in L* value during storage could be attributed to the oxidation of phenolic compounds and other physicochemical phenomena [34], like the formation of adducts or complexes between anthocyanins and quinones, generated throughout the oxidation of polyphenols and the weight loss that occurs during storage [39].

### 3.3. Soluble Solids and Total Phenolic Content

The soluble solid (SSC) as well as total phenolic content (TPC) of control and treated samples is shown in Table 3. As can be seen in Table 3, a significant difference was found in blackberries’ soluble solid content, regarding the value at the beginning of the study (8.93 ± 0.12 °Brix), under storage time with the different packaging treatments.

Overall, SSC content in all treatment groups increased during storage, showing higher values that ranged from 9.73 ± 0.38 °Brix for PLA/β-CD-carvacrol 5.0% to 11.01 ± 0.17 °Brix for PLA/β-CD-thymol 2.5% on day 14, with the exception of packaging control PLA/C, which decreased SSC (8.10 ± 0.10 °Brix).

Similar compositional changes have been described by Forney et al. [40], with an increase in sugar concentration from about 2.2–3.2% as berry ripening progresses. However, a trend change was observed on day 21 of storage, and a slight decrease in SSC was observed, showing that at the end of the study, all PLA packaging treatments maintained the SSC content in blackberries compared to commercial clamshell.

Regarding SSC of raspberries at the beginning of the study (11.80 ± 0.26 °Brix), at day 21, the SCC in PLA/β-CD-thymol 2.5%, PLA/β-CD-thymol 5.0%, PLA/β-CD-carvacrol 2.5%, and PLA/β-CD-carvacrol 5.0% active packaging decreased by 15.5%, 14.7%, 15.0%, and 10.8%, respectively, which was slightly lower than that obtained for packaging control PLA/C (17%), and considerably lower than that obtained for commercial clamshell (22.3%). This shows that all PLA active packaging, and specifically the PLA/β-CD-carvacrol 5.0%, maintained a sustained SSC loss through 21 storage days. As has been described in the literature using coating films containing microcapsules of oregano essential oil to preserve fresh purple yam [41], our results indicated that PLA active packaging containing microcapsules of carvacrol or thymol effectively slowed down the metabolism of berries.

Regarding total phenolic content (TPC), although it decreased gradually in all groups with storage time (see Table 3), the PLA groups containing carvacrol and thymol microcapsules showed a less pronounced decline. The control groups, both the commercial clamshell and PLA/C, had the largest TPC decrease in berry samples. For blackberries, with a TPC initial value of 101.01 ± 6.66 mg gallic acid/100 g weight, it decreased up to 58.07 ± 0.76 mg gallic acid/100 g weight on day 21 in commercial clamshell, which represents a 43% TPC loss with respect to its initial value; while the PLA/β-CD-carvacrol 5.0% packaging revealed 83.09 ± 3.40 mg gallic acid/100 g weight on day 21 of storage, with only a 17.74% loss of TPC, suggesting a slower decline process than that observed in the control group. Concerning the TPC initial value for raspberries (119.10 ± 3.09 mg Gallic acid/100 g weight), the best results at the end of the study were obtained again for the PLA/β-CD-carvacrol 5.0% packaging (116.47 ± 2.58 mg gallic acid/100 g weight), which saw a loss of 2.21%, much lower than that determined for commercial clamshell (34% TPC loss), which showed the largest decrease. This slow decline was attributed to the presence, inside the PLA packages, of free carvacrol or thymol compounds, which exert a marked antioxidant effect, protecting phenolic compounds present in berries from oxidation due to their delocalized electron system and aromatic structure—results that agree with those previously described by Ramos et al. [42].

### 3.4. Headspace Analysis

**Figure 4:**
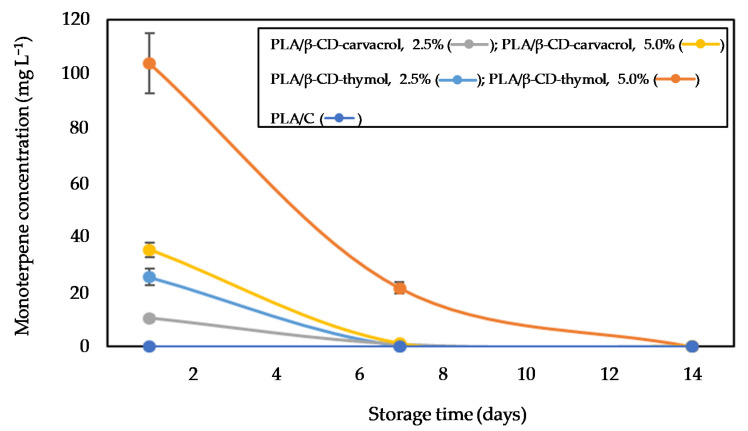
Carvacrol and thymol concentrations in mg L^−1^ (ppm) in the headspace of PLA packages (see inset legend in the figure), by GC-MS at 0, 7, 14, 21 days of storage at 4 °C. Data points represent averages and standard deviations of three replicate samples.

Determination of volatile compounds from carvacrol and thymol was monitored by head space with gas chromatography–mass spectrometry (HS-GC-MS). At each sampling time, the syringe was introduced through the corresponding package septum, withdrawing 1 mL of fluid gas, and immediately released in a splitless injector at 250 °C, obtaining the results showed in Figure 4, corresponding to three replicates for each group and sampling day.

At the initial stage, PLA/β-CD-thymol 5.0% packaging showed the higher volatile concentration, 75% higher than that determined for PLA/β-CD-carvacrol 5.0% and PLA/β-CD-thymol 2.5% packaging. As the storage time was prolonged, only PLA/β-CD-thymol 5.0% packaging maintained a controlled release of monoterpene (30% of initial level at day 7), decreasing asymptotically until the end of the trial, whereas for the rest of the PLA active packaging, the monoterpene levels started to decrease much more quickly until reaching the baseline, with no concentration of any of them detected from the first week of study. A similar behavior was reported by Higueras et al. [43], using films of chitosan containing carvacrol cyclodextrin complexes, evidencing a fast release of carvacrol after the first three days of storage, the concentration of which was maintained over time since packages were hermetically sealed, providing an infinite barrier to carvacrol.

However, in our case, the packages were not hermetically sealed, and the gases could be released outside or a proportion could be absorbed by the fruits without altering their organoleptic properties, as was described by Higueras et al. [43], who argued that the large amount of antimicrobial absorbed or that reacted with the sample caused an unacceptable sensory deterioration.

This release mechanism, observed for carvacrol and thymol in the PLA package, modified the initial atmosphere composition inside the packages, improving the quality and postharvest shelf life of blackberries and raspberries stored under controlled temperature.

### 3.5. Microbiological Quality

**Figure 5:**
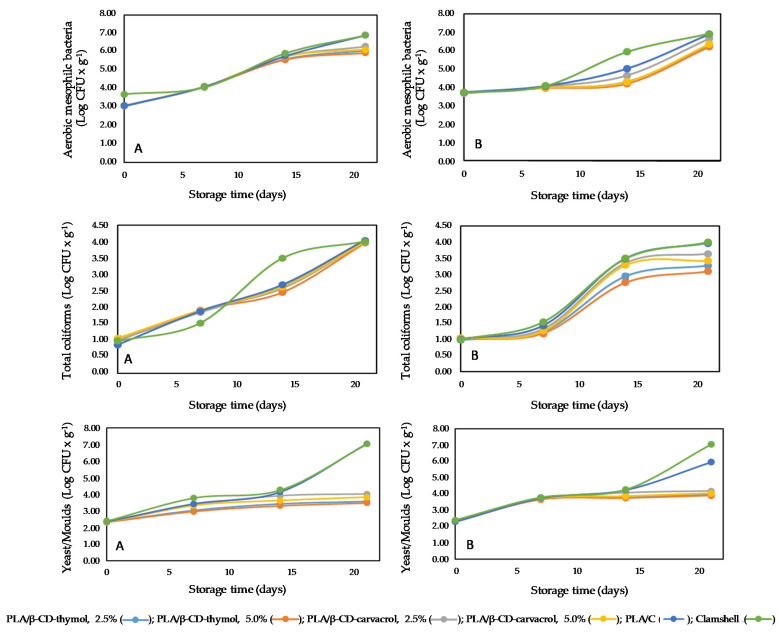
Effect of the type of packaging and storage time (0, 7, 14, 21 days) at 4 °C on the grow of aerobic mesophilic bacteria, totals coliforms, and yeast and molds in blackberries (**A**) and raspberries (**B**).

Microorganisms counts allow for studying the spoiling degree of a particular food during a determined postharvest period. Figure 5 shows the count for aerobic mesophilic bacteria (AMB), total coliforms (TC), and yeasts and molds (YM) for blackberry (Figure 5A) and raspberry (Figure 5B), respectively.

As can be seen in Figure 5A for blackberry samples, a sustained increase is observed in the AMB count during the first two weeks under refrigerated storage conditions, from 1 to approximately 2.5 log *cfu*, independently of the packaging type and concentration of monoterpene, with a marked increase noticeable for both control samples (PLA/C and clamshell), showing at the end of the study a 1 log higher value than that obtained for PLA containing microencapsulates of carvacrol and thymol. Regarding the AMB count for raspberries (Figure 5B), a marked increase for clamshell control samples was observed between the first and the second sampling week (a 2 log *cfu* increase), which was 1.5 units higher than the value obtained for PLA/C, and no appreciable AMB was detected growing in PLA/β-CD-carvacrol 5.0%, PLA/β-CD-thymol 5.0%, or PLA/β-CD-thymol 2.5% packaging. For these same types of packaging, the AMC value increased 2.0 log *cfu* at the end of the study (being 1.5 log *cfu* lower that the values obtained for both control samples).

Coliform growth for blackberries showed a slow increase (close to 0.8 log units) for all PLA packaging materials after seven trial days, whereas this increase was lower by half in clamshell control samples (Figure 5A). However, at day 14, it reached a value of 3.5 log *cfu*, which was higher by 1 log unit compared to the level achieved in all PLA samples, with a consensus value for all groups after three weeks of assay, which was 3 log units higher that the initial value. Regarding coliform growth for raspberries (Figure 5B), in general, a delay was observed through the first week of the test, with better inhibition results for coliforms obtained with PLA/β-CD-thymol 5.0% and PLA/β-CD-thymol 2.5% packaging, both at 14 and 21 days of trial, and it was approximately 0.5 log units lower than the values achieved with the PLA packaging containing microencapsulates of carvacrol at the same percentages.

On the other hand, yeast and molds growth was stopped in raspberries after the first week with all PLA packages containing microencapsulates of carvacrol or thymol (Figure 5B), showing a reduction of 2.2 and 3.2 log units for PLA/C and clamshell control samples, respectively. For blackberries, the growth curve was first low, then gradually increased up to day 14, and subsequently remained constant up to the end of the study for PLA/β-CD-thymol/carvacrol packages (Figure 5A), achieving a slightly better antifungal activity for PLA/β-CD-thymol 5.0% and PLA/β-CD-thymol 2.5% than for PLA packaging containing microencapsulates of carvacrol at the same percentages, and 4.0 log units lower than PLA/C and clamshell control samples, with the berries still fit for consumption. These results are in agreement with those previously described for *Alternaria alternata*, achieving a complete inhibition with PLA packages containing 2.5% and 5% β-CD-carvacrol or 5% β-CD-thymol (wt%) after 10 days of incubation [20], and those obtained against yeasts and molds with PLA films containing 5% and 10% of oregano essential oil in ready-to-eat salads, allowing for the extension of its commercial life [44].

It has been described in the literature that thymol displays a bacteriostatic effect on a wide range of different bacteria and fungi, for which this compound interacts with the lipids on the cell membrane, causing permeabilization and ion leaking, and thus, death [17,26], slightly improving its antimicrobial activity (see Figure 5) as thymol concentration increases [45]. Carvacrol has been reported to present a lower minimum inhibitor concentration (MIC) for different bacterial strains than other phytochemicals, and its synergistic activity with antibiotics has been previously described [46]. This compound exhibits a bacteriostatic effect on microorganisms, with a similar mechanism exerted by thymol. Iron (II) chelating properties of both compounds have been previously described [45,47]; this activity has importance in lipid oxidation reactions, which could explain the inhibition depicted in this study. In addition, the activity displayed for both berries could be related with the amounts of phenolic compounds determined (see Table 3.)

### 3.6. Sensory Atributes

**Figure 6:**
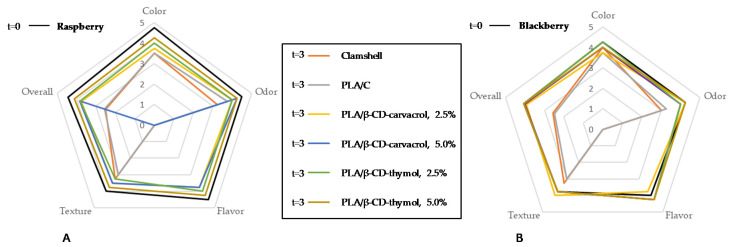
Effect of the type of packaging and storage time (0, 7, 14, 21 days) at 4 °C on sensory attributes of raspberries (**A**) and blackberries (**B**).

The flavor is one of the most valuable sensory attributes in berries for consumers. As can be seen in Figure 6, after 21 days of storage at 4 °C, both products reveal a texture decrease as storage days increased. The highest flavor scores obtained at the end of the study were for samples packed in active PLA packaging containing carvacrol and thymol, and adverse aroma or flavor notes of thymol and carvacrol were not noticed by trained referees. The highest acceptance value (4.25 degrees of acceptance at 21 days) for both berries was assigned to PLA packages containing 5% β-CD-carvacrol, which was the same as that obtained for the control sample at the beginning of the study.

The blackberries and raspberries contained in the clamshell and PLA control packages could not be sensory evaluated at T3, that is, after 21 days of storage, as they showed decomposition and severe fungal attack. Raspberries’ and blackberries’ aroma scores increased during storage due to ripening processes, while a reduction of the texture parameter value was observed. Fruit control color showed 1.5 less degrees of acceptance compared to the fruits in packages containing thymol and carvacrol cyclodextrin complexes. In addition, fruit packaged with both compounds retained the highest degree of color acceptance, with slightly better values for the packages containing thymol. Therefore, the packages that showed the highest overall scores after 21 days of storage at 4 °C for the flavor, odor, color, and texture parameters were, in this order: PLA/β-CD-thymol 5.0%, PLA/β-CD-thymol 2.5%, and PLA/β-CD-carvacrol. These results are in accordance with those previously described in the literature by Viacava et al. [48], since thymol and carvacrol delayed the physiological processes of the fruit and reduced the loss of quality.

## 4. Conclusions

In this work, active PLA packages filled with thymol or carvacrol complexed in β-cyclodextrins (β-CDs) were prepared to evaluate their potential uses to improve berries’ preservation. The results obtained showed the effectiveness of these monoterpenes in improving the shelf life of these fruits. When comparing the results obtained using the developed active PLA packaging materials with control samples along 21 days of storage at 4 °C, a visible growth of microbiological load (mainly yeast and molds) and deterioration in berries’ sensory parameters were observed in control samples, which were higher than those obtained for PLA packaging containing both carvacrol and thymol. The PLA packages containing 5% β-CD-thymol showed a higher inhibition of yeasts and molds (51.57%), with a very good sensory score, with the berries still fit for consumption. Consequently, these results indicated the advantages and potential uses of these biodegradable active packages, to replace the conventional plastics and also the direct addition of preservatives in food formulations, in consonance with bioeconomy principles. The PLA packages containing 5% β-CD-thymol or 5% β-CD-carvacrol showed a promising potential to improve the food quality and safety of berries during a storage period of 21 days at 4 °C, extending their shelf life by one more week, compared to the packages currently used in market. However, more studies are also needed regarding the the durability of the packaging, and to evaluate if these polymers can be reusable several times and for different practical applications.

## Figures and Tables

**Table 1:** Changes in color parameters (L*, a*, b*) of blackberries under different packaging at 0, 7, 14, and 21 days of storage at 4 °C.

Day	Packaging	L*	a*	b*
0	-	17.91 ± 0.48 ^a^	0.82 ± 0.13 ^a^	−0.93 ± 0.11 ^a^
7	Clamshell	15.65 ± 0.42 ^bc^	1.35 ± 0.59 ^b^	−0.91 ± 0.08 ^a^
	PLA/C	15.98 ± 0.27 ^bc^	3.35 ± 0.59 ^b^	−0.48 ± 0.23 ^b^
	PLA/β-CD-thymol, 2.5%	15.28 ± 0.99 ^bc^	1.67 ± 0.29 ^a^	−1.01 ± 0.03 ^a^
	PLA/β-CD-thymol, 5.0%	18.23 ± 0.16 ^a^	1.13 ± 0.11 ^ab^	−0.95 ± 0.07 ^a^
	PLA/β-CD-carvacrol, 2.5%	18.23 ± 0.27 ^a^	1.32 ± 0.36 ^ab^	−0.91 ± 0.08 ^a^
	PLA/β-CD-carvacrol, 5.0%	17.01 ± 1.07 ^ab^	0.92 ± 0.22 ^a^	−1.08 ± 0.30 ^a^
14	Clamshell	13.02 ± 0.76 ^c^	1.05 ± 0.10 ^ab^	−0.55 ± 024 ^b^
	PLA/C	14.02 ± 0.76 ^c^	2.05 ± 0.3 ^b^	−0.57 ± 0.20 ^b^
	PLA/β-CD-thymol, 2.5%	17.26 ± 0.87 ^ab^	0.68 ± 0.16 ^a^	−1.11 ± 0.04 ^a^
	PLA/β-CD-thymol, 5.0%	16.12 ± 0.30 ^bc^	1.09 ± 0.10 ^ab^	−1.02 ± 0.03 ^a^
	PLA/β-CD-carvacrol, 2.5%	15.20 ± 0.37 ^c^	1.75 ± 0.74 ^ab^	−0.55 ± 0.04 ^b^
	PLA/β-CD-carvacrol, 5.0%	15.99 ± 0.21 ^bc^	0.74 ± 0.03 ^a^	−1.06 ± 0.08 ^a^
21	Clamshell	12.92 ± 0.36 ^c^	0.66 ± 0.18 ^a^	−0.55 ± 024 ^b^
	PLA/C	13.65 ± 0.42 ^bc^	2.05 ± 0.63 ^b^	−0.57 ± 0.20 ^b^
	PLA/β-CD-thymol, 2.5%	17.10 ± 0.77 ^ab^	0.63 ± 0.21 ^a^	−1.15 ± 0.10 ^a^
	PLA/β-CD-thymol, 5.0%	16.10 ± 0.29 ^bc^	1.12 ± 0.15 ^ab^	−1.11 ± 0.15 ^a^
	PLA/β-CD-carvacrol, 2.5%	14.90 ± 0.80 ^c^	1.72 ± 0.74 ^ab^	−0.73 ± 0.34 ^ab^
	PLA/β-CD-carvacrol, 5.0%	15.95 ± 0.27 ^bc^	0.68 ± 0.08 ^a^	−1.10 ± 0.08 ^a^

* Values are reported as average ± standard deviation of triplicate determinations. Values in the same column with different letters are significantly different statistically (*p* < 0.05).

**Table 2:** Changes in color parameters (L*, a*, b*) of raspberries under different packaging at 0, 7, 14, and 21 days of storage at 4 °C.

Day	Packaging	L*	a*	b*
0	-	28.26 ± 0.68 ^a^	26.49 ± 1.17 ^a^	10.85 ± 1.09 ^a^
7	Clamshell	25.29 ± 0.90 ^c^	24.65 ± 0.34 ^a^	8.01 ± 0.24 ^b^
	PLA/C	27.46 ± 1.34 ^ab^	28.10 ± 1.23 ^ab^	8.05 ± 0.09 ^b^
	PLA/β-CD-thymol, 2.5%	27.80 ± 1.26 ^ab^	31.57 ± 2.18 ^b^	8.52 ± 0.37 ^b^
	PLA/β-CD-thymol, 5.0%	27.39 ± 0.90 ^c^	25.78 ± 0.33 ^ab^	8.93 ± 0.59 ^b^
	PLA/β-CD-carvacrol, 2.5%	28.48 ± 0.09 ^a^	25.44 ± 0.52 ^ab^	8.77 ± 0.24 ^b^
	PLA/β-CD-carvacrol, 5.0%	28.35 ± 0.65 ^ab^	25.26 ± 1.99 ^ab^	8.76 ± 0.56 ^b^
14	Clamshell	24.08 ± 0.17 ^b^	21.20 ± 1.32 ^b^	6.77 ± 0.39 ^b^
	PLA/C	25.73 ± 0.52 ^c^	23.01 ± 0.23 ^ab^	6.87 ± 0.45 ^b^
	PLA/β-CD-thymol, 2.5%	27.17 ± 0.16 ^ab^	24.29 ± 0.34 ^a^	7.21 ± 0.87 ^b^
	PLA/β-CD-thymol, 5.0%	26.92 ± 0.23 ^b^	24.51 ± 0.74 ^a^	8.13 ± 0.51 ^b^
	PLA/β-CD-carvacrol, 2.5%	26.33 ± 0.59 ^ab^	22.45 ± 1.49 ^a^	7.90 ± 0.80 ^b^
	PLA/β-CD-carvacrol, 5.0%	26.53 ± 0.21 ^bc^	21.83 ± 2.28 ^b^	7.54 ± 0.15 ^b^
21	Clamshell	23.96 ± 0.54 ^b^	16.57 ± 0.50 ^c^	6.11 ± 0.28 ^b^
	PLA/C	25.57 ± 0.59 ^b^	22.08 ± 2.80 ^a^	6.35 ± 0.45 ^b^
	PLA/β-CD-thymol, 2.5%	26.29 ± 0.34 ^b^	22.16 ± 1.71 ^b^	7.09 ± 0.47 ^b^
	PLA/β-CD-thymol, 5.0%	26.78 ± 0.33 ^a^	22.98 ± 1.08 ^ab^	8.03 ± 0.39 ^b^
	PLA/β-CD-carvacrol, 2.5%	25.08 ± 0.17 ^b^	21.91 ± 0.61 ^b^	7.81 ± 0.59 ^b^
	PLA/β-CD-carvacrol, 5.0%	25.23 ± 1.26 ^b^	21.71 ± 0.60 ^c^	7.50 ± 1.25 ^b^

* Values are reported as average ± standard deviation of triplicate determinations. Values in the same column with different letters are significantly different statistically (*p* < 0.05).

**Table 3:** Evolution of soluble solid content (SSC) expressed as Brix grade (°Brix) and total phenolic content (TPC) expressed as milligrams of gallic acid equivalents per 100 g of sample (mg GAE/100 g) of blackberries and raspberries under different packaging at 0, 7, 14, and 21 days of storage at 4 °C.

		Blackberries		Raspberries	
Day	Packaging	°Brix	mg GAE/100 g	°Brix	mg GAE/100 g
0	-	8.93 ± 0.12 ^a^	101.01 ± 6.66 ^a^	11.80 ± 0.26 ^a^	119.10 ± 3.09 ^a^
7	Clamshell	8.67 ± 0.06 ^ac^	62.58 ± 0.51 ^d^	9.70 ± 0.20 ^cd^	80.41 ± 0.43 ^cd^
	PLA/C	8.33 ± 0.23 ^cd^	61.80 ± 1.37 ^b^	9.17 ± 0.06 ^d^	76.55 ± 7.26 ^d^
	PLA/β-CD-thymol, 2.5%	9.67 ± 0.21 ^b^	71.98 ± 13.49 ^c^	9.23 ± 0.35 ^d^	86.08 ± 19.78 ^bcd^
	PLA/β-CD-thymol, 5.0%	9.20 ± 0.35 ^ab^	81.91 ± 22.41 ^ab^	9.13 ± 0.15 ^d^	79.10 ± 10.77 ^abc^
	PLA/β-CD-carvacrol, 2.5%	9.00 ± 0.17 ^a^	80.03 ± 7.79 ^c^	10.33 ± 0.45 ^bc^	110.5 ± 2.51 ^ab^
	PLA/β-CD-carvacrol, 5.0%	8.07 ± 0.15 ^d^	88.35 ± 1.25 ^bc^	10.50 ± 0.10 ^b^	100.88 ± 24.88 ^a^
14	Clamshell	10.97 ± 0.21 ^d^	60.45 ± 0.56 ^d^	9.80 ± 0.10 ^cd^	80.43 ± 1.26 ^b^
	PLA/C	8.10 ± 0.10 ^b^	80.55 ± 5.48 ^bc^	8.53 ± 0.21 ^c^	83.40 ± 4.32 ^b^
	PLA/β-CD-thymol, 2.5%	11.01 ± 0.17 ^d^	92.40 ± 15.71 ^a^	10.27 ± 0.47 ^bc^	69.82 ± 10.54 ^ab^
	PLA/β-CD-thymol, 5.0%	10.50 ± 0.10 ^ab^	92.28 ± 6.34 ^ab^	10.90 ± 0.26 ^ab^	82.52 ± 16.46 ^b^
	PLA/β-CD-carvacrol, 2.5%	10.93 ± 0.25 ^d^	72.83 ± 4.66 ^cd^	9.10 ± 0.69 ^de^	106.09 ± 4.79 ^ab^
	PLA/β-CD-carvacrol, 5.0%	9.73 ± 0.38 ^c^	81.70 ± 10.64 ^bc^	10.63 ± 0.31 ^bc^	103.38 ± 8.54 ^ab^
21	Clamshell	9.57 ± 0.45 ^a^	58.07 ± 0.76 ^c^	9.17 ± 0.57 ^c^	79.30 ± 0.53 ^b^
	PLA/C	8.83 ± 0.15 ^a^	68.59 ± 1.00 ^bc^	9.80 ± 0.44 ^bc^	83.90 ± 13.34 ^b^
	PLA/β-CD-thymol, 2.5%	8.43 ± 1.08 ^a^	57.57 ± 7.07 ^c^	9.97 ± 0.15 ^bc^	100.92 ± 11.86 ^ab^
	PLA/β-CD-thymol, 5.0%	8.83 ± 0.06 ^a^	68.29 ± 2.98 ^bc^	10.07 ± 0.06 ^bc^	102.84 ± 15.37 ^ab^
	PLA/β-CD-carvacrol, 2.5%	8.23 ± 0.21 ^a^	70.27 ± 7.91 ^bc^	10.03 ± 0.12 ^bc^	84.86 ± 11.46 ^ab^
	PLA/β-CD-carvacrol, 5.0%	8.30 ± 0.40 ^a^	83.09 ± 3.40 ^b^	10.53 ± 0.57 ^b^	116.47 ± 2.58 ^a^

* Values are reported as average ± standard deviation of triplicate determinations. Values in the same column with different letters are significantly different statistically (*p* < 0.05).
